# Sub-THz Imaging Using Non-Resonant HEMT Detectors

**DOI:** 10.3390/s18020543

**Published:** 2018-02-10

**Authors:** Juan A. Delgado-Notario, Jesus E. Velazquez-Perez, Yahya M. Meziani, Kristel Fobelets

**Affiliations:** 1 Nano Lab, Salamanca University, Salamanca 37008, Spain; juanandn@usal.es; 2 Department of Electrical and Electronic Engineering, Imperial College, South Kensington Campus, London SW7 2AZ, UK; k.fobelets@imperial.ac.uk

**Keywords:** terahertz, SiGe, MODFET, imaging, plasma wave, detector

## Abstract

Plasma waves in gated 2-D systems can be used to efficiently detect THz electromagnetic radiation. Solid-state plasma wave-based sensors can be used as detectors in THz imaging systems. An experimental study of the sub-THz response of II-gate strained-Si Schottky-gated MODFETs (Modulation-doped Field-Effect Transistor) was performed. The response of the strained-Si MODFET has been characterized at two frequencies: 150 and 300 GHz: The DC drain-to-source voltage transducing the THz radiation (photovoltaic mode) of 250-nm gate length transistors exhibited a non-resonant response that agrees with theoretical models and physics-based simulations of the electrical response of the transistor. When imposing a weak source-to-drain current of 5 μA, a substantial increase of the photoresponse was found. This increase is translated into an enhancement of the responsivity by one order of magnitude as compared to the photovoltaic mode, while the NEP (Noise Equivalent Power) is reduced in the subthreshold region. Strained-Si MODFETs demonstrated an excellent performance as detectors in THz imaging.

## 1. Introduction

The terahertz (THz) region lies in the gap between microwaves and infrared regions of the electromagnetic (EM) spectrum. Across the past 25 years, motivated by the strikingly vast range of possible applications for THz radiation, many new terahertz techniques have been investigated and demonstrated [1]. This vast range of THz sensing applications includes astronomy [2,3], spectroscopy (various rotational, vibrational, and translational modes of light-weight molecules are within the THz range; since these modes are specific to a particular substance, it is possible to obtain a THz fingerprint) [4,5,6], communications with a bandwidth significantly higher than those based on microwaves [7] (detection of high data rate wireless communication has been demonstrated [8]), security based both on imaging of concealed objects and spectroscopy [9], metrology [10], etc. The idea of using terahertz radiation for imaging is based in the unique properties of THz rays (T-rays), since common packaging materials such as cardboard and plastics are optically opaque, but compared to T-rays the detection of concealed objects is, in principle, feasible. Moreover T-rays permit imaging with a diffraction-limited resolution similar to that of the human eye [11]. T-rays can penetrate a few millimeters of skin, hence they can be used in the diagnostics of subcutaneous tissue, for instance, to detect skin cancer in vivo [12]. Nevertheless, further development of sources and detectors is necessary to make THz applications an everyday reality. Solid-state semiconductor devices allow the development of low-cost, compact, and reliable systems; accordingly, the extension of their operation frequency range is of great interest to implement THz sources and detectors.

Plasma wave terahertz electronics is one of the most promising ways to achieve THz detection using solid-state devices at room temperature. Oscillations of the plasma waves in the channel of sub-micron field-effect transistors (FETs) is a means to obtain emission and detection of THz signals that were proposed by Dyakonov and Shur in a pioneering theoretical work [13,14,15]. They predicted that nonlinear properties of two-dimensional (2D) plasma in the sub-micron transistor channel can be used for the detection of THz radiation. Plasma wave-based detectors perform a direct conversion of the power of the incoming EM radiation into voltage or current; the increasing availability of continuous-wave compact sources based on solid-state oscillators in the millimeter-wave range raises the interest on the direct detectors for THz imaging [16]. Plasma waves in gated 2-D electron systems behave like sound waves in a gas. In short gate FETs, the plasma wave frequencies lie in the terahertz range. In particular, Dyakonov and Shur demonstrated that an open-drain FET subjected to an electromagnetic radiation develops a DC drain-to-source voltage, transducing the THz radiation (photovoltaic detection). The value of this voltage is non-linearly modulated by the gate-to-source bias voltage, as this voltage controls the carrier concentration in the transistor’s channel. An additional mechanism to explain broadband detection by FETs is based on distributed self-mixing [17,18]. Detectors based on plasma wave devices present several advantages: low cost, small size, room temperature operation, and tuning of the resonant frequency in the THz range through the gate bias.

An experimental investigation has been then conducted on different types of FETs, demonstrating their capabilities for detection of terahertz radiation. Resonant detection by GaAs/AlGaAs FETs was first reported by Knap et al. [16] at 8 K. Also, in 2002 they reported on non-resonant detection at room temperature [19]. In 2004, room temperature, non-resonant detection by silicon field-effect transistors was demonstrated for the first time, [20], in which the responsivity was estimated at around 200 V/W and the Noise Equivalent Power (NEP) at around 1 pW/√Hz [21,22]. THz imaging based on CMOS technology has been reported by different groups [23,24]. In 2009, an array build using 0.25 µm CMOS technology with responsivity of 80 kV/W and NEP of 300 pW/√Hz that was used for imaging at 0.65 THz was reported [17]. Recently, room-temperature plasmonic detection of sub-terahertz radiation by InAlAs/InGaAs/InP High Electron Mobility Transistors (HEMTs) with asymmetric dual-grating-gate structure exhibiting a responsivity of 22.7 kV/W at 200 GHz in the photovoltaic mode and noise equivalent power of 0.48 pW/√Hz [25,26] has been reported. A review of the state of the art in THz direct detection can be found in [27].

The present work reports on an original study of the modulation of the sub-THz performance of a Π-gate Si/Si_0.7_Ge_0.3_ MODFET by current-biasing the device. An increase by approximately one order of magnitude of the MODFET responsivity at 150 and 300 GHz was measured when a source-to-drain bias-current of 5 μA was imposed. Experimental results were in good agreement with TCAD simulation, which justifies the observed responsivity’s modulation in terms of a channel symmetry introduced by the bias-current. A main distinct interest of the high-mobility n-type FETs based on the Si/SiGe system is that, unlike the one based on the III-V plasmon detectors, it allows for the integration of high-mobility FETs, which are necessary for THz detection based on plasma waves, along with mainstream Si technology, since both are fabricated on conventional Si wafers. This paves the way for monolithic integration in a single chip of a high-performance THz detector and the readout circuitry (basic analog building blocks were already demonstrated using Si/SiGe FETs [28], but conventional bipolar or CMOS circuitry may be fabricated instead) and, accordingly, would promise future low-cost, high-reliability, high-speed, and compact THz sensors. 

The paper is organized as follows: In Section 2 the Si/SiGe MODFET, along with its geometry and epilayer description, is presented; detection of sub-THz [29,30] and imaging using Si/SiGe transistors were already demonstrated [31]. The physics-based TCAD tool based on the method of moments used to simulate the transistor is subsequently presented. In the last part of the section, the experimental setups are presented. In Section 3 results are presented: DC and low frequency AC characteristics show an excellent agreement between experimental and simulation results serving as a validation of the TCAD model developed. Also, a qualitative agreement was found when comparing TCAD results with measurements; both show a broadband detection of the THz radiation previously found in T-gate Si/Ge FETs [29,30,31]. Responsivity and NEP (Noise Equivalent Power) were obtained in the photovoltaic mode. Subsequently, the device responsivity and NEP were determined when the transistor was current-biased using a weak drain-to-source current.

## 2. Materials and Methods

In this section, we will describe the strained-Si MODFETs (Modulation-Doped FETs) used as THz detector in the present work. The measurement techniques and the experimental setup will be presented together with the theoretical models used in simulations to analyze de strained-Si FETs.

### 2.1. Si/SiGe MODFET

As pointed out above, high-mobility short channels are necessary to achieve plasma-wave THz detectors. The material system Si/SiGe allows for the creation of a thin layer of strained silicon under tetragonal (biaxial tensile) strain. This strain has significant implications for the band structure of the semiconductor and a high impact on the performance of the MODFET (Modulation-Doped Field-Effect Transistor) devices that are based on the Si/SiGe system. Tetragonal strain has the effect of lifting the six-fold degeneracy of the conduction band in silicon into two-fold and four-fold degenerate sets, thus lowering the energy of the two valleys with their long axis perpendicular to the Si/SiGe interface. Since intervalley carrier scattering may only occur between degenerate minima, electrons in a layer of (tensile) strained silicon would undergo a lower number of intervalley scattering events per unit time than in bulk silicon. The combination of lower scattering rates (that is, higher values of the momentum relaxation time) and a lower value of the electron conductivity mass as compared to bulk Si make tensile strained silicon layers excellent candidates to build the high-mobility plasma in the FET channel that is necessary to detect THz radiation.

**Figure 1:**
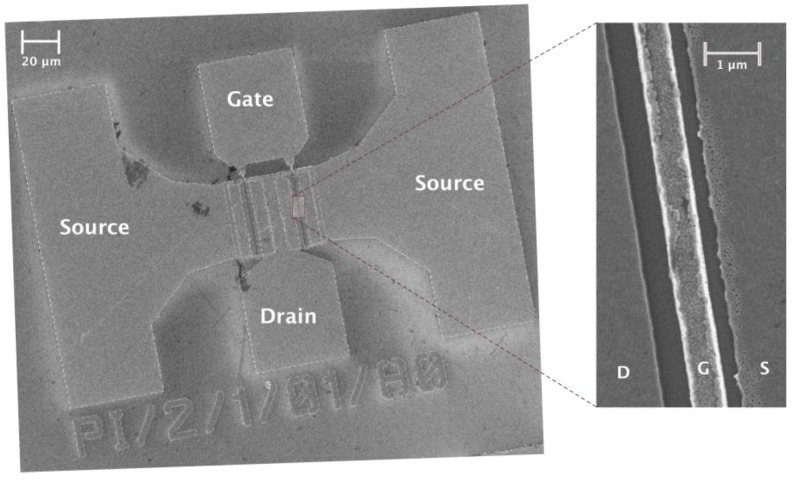
(**Left**) SEM image of a Π-gate Si/SiGe MODFET under study; (**Right**) Zoom detailing the top view of the lateral structure of the metal gate, drain and source contacts. The Schottky-gate is placed in a slightly asymmetric position between the source and the drain on the device channel.

**Figure 2:**
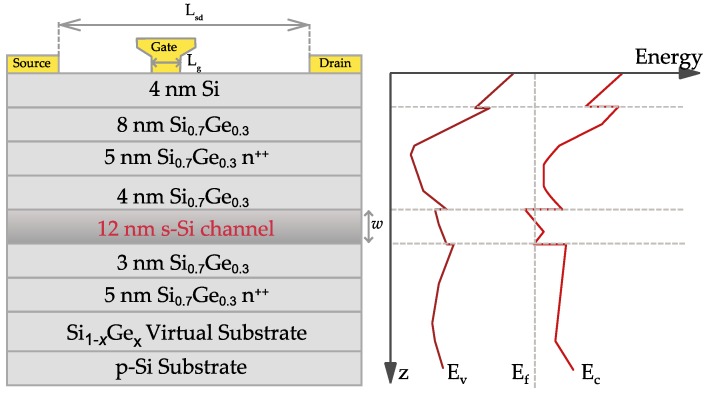
(**Left**) Cross section of the Si/SiGe MODFETs showing the vertical layout of the transistor with a schematic of the contacts; the strained-Si layer of thickness *w* is highlighted. (**Right**) A plot of the vertical profiles of both bands edges and the Fermi level under the gate in equilibrium is given; the double-deck supply layer structure leads to a double electron channel in the quantum well [32].

A SEM image of the Si/SiGe MODFET is given in Figure 1. The vertical layer structure of the transistor along with its energy band diagram at equilibrium is presented in Figure 2. The value of the conduction band offset of the heterojunction Si/Si_0.70_Ge_0.30_ is about 180 meV, ensuring an excellent electron confinement in the strained-Si quantum well layer that is necessary for room temperature high-mobility operation of the detector.

A linearly graded virtual substrate was grown on a high resistivity p-type Si substrate. Molecular beam epitaxy (MBE) was subsequently employed to grow the layers of the heterostructure shown in Figure 2. The highlighted layer in Figure 2 with a thickness *w* = 12 nm represents the strained quantum well sandwiched between the two Si_0.70_Ge_0.30_ relaxed layers. Two Sb-doped supply layers provide the carriers to the strained-Si quantum-well channel [32,33]; to ensure high channel mobility spacers were used to reduce remote impurity scattering by dopants in the supply layers. The source and drain ohmic contacts were not self-aligned. Pt/Au was used for the Schottky-gate that is not symmetrically placed between source and drain (Figure 1 and Figure 2). The total drain-to-source distance, *L_sd_*, is 2 μm, the gate length *L_g_* = 250 nm, and the gate width is 60 μm. The gate is asymmetrically placed between the source and the drain contacts, and the distance between the right edge of the source and the left edge of the gate is 1 μm. The asymmetrical position of the gate is of interest to enhance THz detection by the transistor.

### 2.2. Technology-CAD (TCAD) Simulation

In their seminal work, Dyakonov and Shur used the Euler equation for one space dimension to demonstrate the ability of plasma-waves FETs to generate and detect THz radiation. While a one-dimensional equation is suitable to obtain an analytic model and to draw general conclusions about the frequency response of a simplified ideal FET, it is not enough to describe accurately its electrical behavior [13,14,15]. Several aspects such as complex doping profiles, non-scalar effective carrier masses, scattering time dependent on the carrier energy, high electric fields that locally modify the carrier mobility, and bi-dimensional structure of the devices, etc., cannot be included in a single equation. 

A better description of the charge transport in a transistor may be achieved using the drift-diffusion model (DDM) that consists of the Poisson equation and the continuity equations for electrons and holes:

(1) $$\nabla^{2}\varphi =-\frac{q}{\varepsilon }\left(p-n+N_{D}^{+}-N_{A}^{-}\right),$$

(2) $$\frac{\partial n}{\partial t}=\frac{1}{q}\left(\vec{\nabla }\cdot \vec{J_{n}}\right)-U_{n},$$

(3) $$\frac{\partial p}{\partial t}=-\frac{1}{q}\left(\vec{\nabla }\cdot \vec{J_{p}}\right)-U_{p},$$

in which *ϕ* is the electric potential, *q* is the absolute value of the electron charge, *n* (*p*) is the electron (hole) concentration, $N_{D}^{+}$ ($N_{A}^{-}$) is the ionized donor (acceptor) concentration, *ε* is the local material permittivity, ${\vec{J}}_{n}$ (${\vec{J}}_{p}$) is the current density of electrons (holes) in the drift-diffusion model, and *U_n_* (*U_p_*) represent the net electron (hole) recombination rate. In deep-submicron FETs, the drain and gate bias give rise to large electric fields that rapidly change over small length scales, giving the lead to nonlocal phenomena that dominate the transistor performance [33]. The strong carrier heating by the intense electric fields in the channel of a FET leads to carrier temperatures significantly larger than the lattice one. Accordingly, energy balance equations accounting for electron and hole heating and energy relaxation must be self-consistently added to the transport model. Since the DDM only takes into account moment relaxation [34], it is unable to correctly describe hot carrier transport, and it becomes invalid for short-channel FETs. As channel mobility is closely dependent on the carrier temperature in these devices, a new extended model needs to be used to study the electric properties of deep-submicron FET transistors used in plasma wave THz detection. This extended model is known as the hydrodynamic model (HDM).

The HDM [34,35], includes carrier energy balance by coupling to the set of Equations (1)–(3), the electron, and hole energy flow densities that are given as:

(4) $$\vec{\nabla }\cdot \vec{S_{n}}=\frac{1}{q}\vec{J_{n}}\cdot \vec{E}-\frac{3}{2}\left(n\frac{u_{n}-u_{0}}{\tau_{n}}+\frac{\partial \left(u_{n}n\right)}{\partial t}\right),$$

(5) $$\vec{\nabla }\cdot \vec{S_{p}}=\frac{1}{q}\vec{J_{p}}\cdot \vec{E}-\frac{3}{2}\left(p\frac{u_{p}-u_{0}}{\tau_{p}}+\frac{\partial \left(u_{p}p\right)}{\partial t}\right),$$

in which $\vec{S_{n}}$ ($\vec{S_{p}}$) is the electron (hole) energy flux, *τ_n_* (*τ_p_*) is the electron (hole) energy relaxation time, *u_n_* (*u_p_*) and *u*_0_ are the electron (hole) and lattice thermal voltages, and $\vec{E}$ the electric field that is self-consistently obtained from the Poisson equation.

To reduce the computational burden, taking into account that the strained-Si MODFET is essentially a majority carrier device, only the electron energy balance was considered. In the present work, a two-dimensional HDM was used to obtain the photovoltaic THz response of the strained-Si MODFETs using Synopsys TCAD [36] simulation software. Electron and hole energy relaxation times are usually obtained from uniform-field Monte Carlo simulations [37,38]. In TCAD simulations impurity de-ionization, Fermi-Dirac statistics and mobility degradation due to both longitudinal and transverse electric field were taken into account. All TCAD simulations were carried out at room temperature. As TCAD simulations were two-dimensional, the obtained currents are given in A/m.

The TCAD simulations carried out, as it was pointed out above, were 2D. The geometry and dimensions used in the simulations were the ones shown in Figure 2 (left). The doping level of the supply layers was: 10^19^ cm^−3^ for the upper supply layer and 2.5 × 10^18^ cm^−3^ for the lower one. The thickness of the virtual substrate was 600 nm and the p-substrate, with a doping level of 10^16^ cm^−3^, was assumed to have a thickness of 500 nm in order to save computer memory. In the remaining regions, non-intentionally doped, a residual uniform doping of 10^15^ cm^−3^, was assumed in simulations. Both source and drain contacts were placed on implanted, highly doped regions to ensure low values of their contact resistance. Reference [39] presents an extensive review work about the Si/SiGe material systems; in particular, the value of the conduction and valence bands offsets between the strained-Si and the relaxed Si_1−x_Ge_x_ as a function of the Ge molar fraction (x = 0.3) were extracted from this reference. The conduction band offset (about 180 meV as above mentioned) is a key parameter to ensure that electrons are not transferred from the quantum well into the surrounding low-mobility regions. Low electric field mobility in the channel was modeled using the Roldan’s model for biaxially-strained Si on relaxed SiGe [40], and the maximum value electron mobility in the channel was 1300 cm^2^/(V⋅s). For high electric fields, the mobility degradation is modeled by combining the Caughey-Thomas empirical model [41] and the effective-field carrier temperature model [36]. The energy relaxation time was taken equal to 0.2 ps from [42]. Finally, electric permittivity in the strained Si layer was assumed to be equal to the one in relaxed Si, and in the SiGe layers it was obtained by linear interpolation of the low-frequency electric permittivity values of Si and Ge.

### 2.3. Terahertz Measurements and Imaging

**Figure 3:**
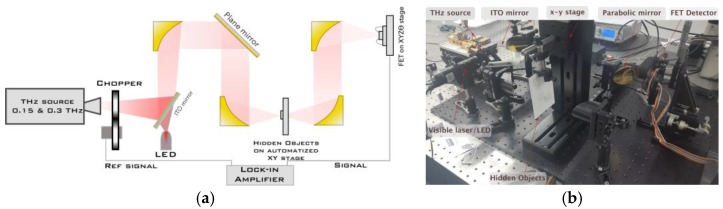
(**a**) Schematic description of the detection and imaging experimental setup: the THz source generated two output frequencies 150 and 300 GHz; (**b**) photograph of the experimental setup. On the upper left corner the source by RPG can be seen; in the center, the automated x-y stage is shown (the sample is placed inside the envelope when the system is used for imaging).

The schematic and a photograph of the experimental setup used for terahertz characterization of the detector and imaging are shown in Figure 3. A solid-state harmonic generator sub-THz source based on a DRO with output power of 6 mW at 300 GHz and 3 mW at 150 GHz was used to excite the device. The emitted power was measured close to the output of the source using a highly sensitive calibrated pyroelectric detector. The incoming THz radiation was modulated by a mechanical chopper between 0.233 to 5 kHz, collimated and focused by off-axis parabolic mirrors. A red LED (or laser) in combination with an indium tin oxide (ITO) mirror was used for the alignment of the THz beam. All THz measurements were carried out at room temperature.

When the setup is used to measure the THz response of the detector, the x-y stage is not used; the Si/SiGe MODFET under study was glued and wire-bounded on a dual in line package (DIP8). No special attached antennas were used in the experiment; therefore, the terahertz radiation was coupled to the device through bonding wires and/or metallization contact pads. The photo-induced drain-to-source voltage, *ΔU*, was measured using a lock-in technique. A Stanford Research SR830 lock-in amplifier with a 10 MΩ input impedance was used to measure *ΔU*. The image is generated pixel-by-pixel using the x-y stage.

Standard DC and AC electrical of the transistors were realized on wafer using an Agilent B1500A semiconductor Device Parameter Analyzer (Agilent, Hachioji, Japan) and a Cascade Summit 11000B-AP (Cascade Michotech, Beaverton, OR, USA). All electrical measurements were carried out at room temperature.

## 3. Results and Discussion

### 3.1. Electrical Characterization

**Figure 4:**
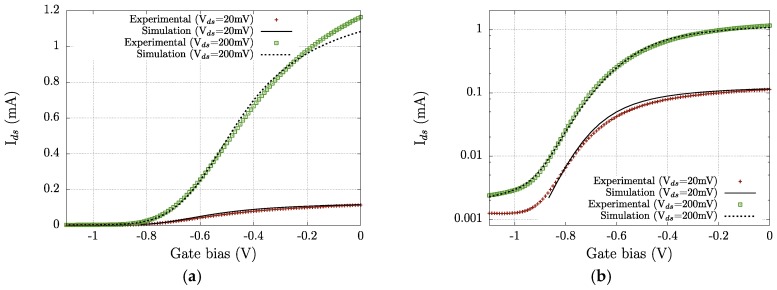
(**a**) Experimental and simulated transfer characteristics of the strained-Si MODFET for two values, 20 mV and 200 mV, of the drain voltage; (**b**) experimental and simulated transfer characteristics with current plotted in log scale.

The transfer characteristics of the strained-Si MODFET are given in Figure 4 for two different values of the drain to source voltage (*V_ds_*). The transistors are depletion-mode devices and a negative bias voltage must be applied to the gate (*V_gs_*< 0) to cut-off the channel [28,43]. Transfer characteristics in the log-scale (Figure 4b) show that a total switch-off of the device is not possible, as a significant level of drain current (*I_ds_*) persists for a gate bias of −1.2 V, i.e., in the subthreshold region (*V_gs_* < *V_th_*) a noticeable drain-to-source leakage current is present. As the drain voltage is moderately raised from 20 mV to 200 mV, the above described behavior is enhanced and the sub-threshold current at *V_gs_* = −1.2 V increases when *V_ds_* increases. As above pointed, this behavior reveals a moderate control of the channel by the gate electrode due to the double deck structure of the supply layers; in return, this double deck ensures a suitable concentration of the electron plasma in the channel that is of paramount importance to achieving a good performance of the transistor in THz detection.

**Figure 5:**
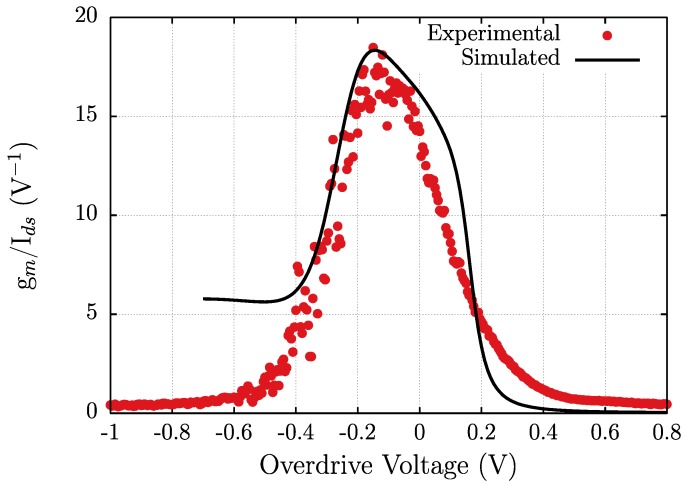
Efficiency of the transconductance vs. the gate overdrive voltage calculated from measurements and numerical TCAD simulations at *V_ds_* = 20 mV.

The TCAD simulation structure is as follows: first, for each gate-to-source bias value, the two-dimensional set of equations of the HDM is solved using the proper boundary conditions, and the internal magnitudes of the transistor at the bias point are determined; then, time-domain simulations are conducted varying the superimposed small signal on the gate electrode; the time step is adapted to the value of the sub-THz frequency of the signal to ensure a correct sampling of the sinusoid [44]. The TCAD simulation model of the transistor was validated through comparison with DC and AC measurements. In Figure 4, the experimental and calculated transfer characteristics for *V_ds_* = 20 mV and *V_ds_* = 200 mV are given. Figure 5 shows the efficiency of the transconductance versus the gate overdrive (*V_gs_*−*V_th_*) of the strained-Si MODFET under study. Loading effect in measurements can be excluded (even when the device is biased in the weak inversion region), as the channel resistance is always below 20 kΩ (3 orders of magnitude lower than the input impedance of the lock-in amplifier).

The DC agreement between the TCAD and the experimental results across the whole range of the gate-to-source bias is excellent. The efficiency of the transconductance, defined as the ratio of transconductance (*g_m_*) to drain-to-source DC current (*I_ds_*), is a key parameter conventionally used to compare the performance of different technologies of transistors, and it is strongly related to the design of basic building blocks in analog circuits. The efficiency of the transconductance is used here, on the one hand, because it clearly shows the operation region of the device and, on the other hand, since the efficiency of the transconductance is linearly dependent on a current derivative, discrepancies between simulation and experimental results are readily revealed. Figure 5 shows that TCAD results acceptably follow measurements for values of the overdrive voltage above −0.4 V. Since plasma wave THz detection needs a plasma channel that is well established, the discrepancies found between TCAD and experimental results for gate overdrive voltages below −0.4 V are not relevant to the analysis of THz detection by plasma waves, as the transistors cannot operate as THz detector for very negative overdrive voltages. The maximum value of the efficiency of the transconductance, which is significantly lower than the theoretical limit (38 V^−1^), along with the above discussed behaviour of the transconductance, suggests that further improvements of the transistor performance may be achieved by the optimization of the layout of the structure. 

### 3.2. Terahertz: TCAD and Experimental Characterization. THz Imaging

The TCAD study of the THz photovoltaic response of the transistor was implemented, as in measurements, grounding the source, biasing the gate, and floating the drain contact, while a sub-THz sinusoidal signal is superimposed to the gate voltage as described in references [13,14,15]. An electromagnetic solver, [45], along with the HDM, would be necessary in order to fully simulate the electromagnetic coupling of the incoming THz wave to the electron channel; given the numerical complexity of self-consistently solving the HDM model coupled to the Maxwell equations, the present work is limited to the use of the HDM model assuming an arbitrary value of the sub-THz amplitude on the gate. The amplitude of the gate signal was fixed to 5 mV for both 150 and 300 GHz. Since the amplitude of the AC gate voltage is arbitrary, the magnitude of the THz response will be presented as arbitrary units in figures. 

Below we will remark that, as a consequence of the absence of the electromagnetic solver, the qualitative matching between measurements and TCAD results at 150 GHz is poorer than at 300 GHz. In TCAD simulations, it was found that the drain voltage (*ΔU*) induced by the THz sinusoids exhibits both the same shape (sinusoidal) and frequency as the AC one superimposed to the gate bias; this ensures that no frequency conversion takes place. Additionally, it was found that its amplitude is considerably smaller than the one of the gate’s signal, which is in agreement with the fact that in the sub-THz range the transistor is unable to amplify signals. The mean value of the induced drain voltage was negative, in good agreement with theoretical models [13,14,15].

**Figure 6:**
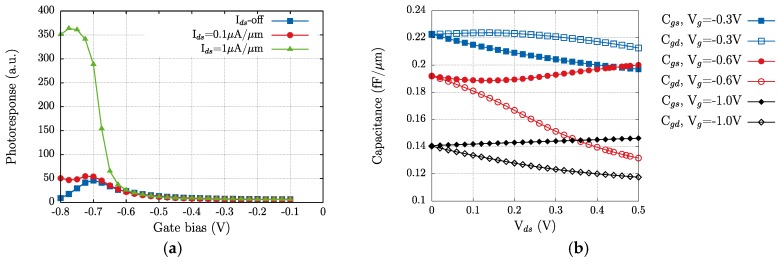
(**a**) Calculated THz photoresponse of the strained-Si MODFET in the photovoltaic mode (*I_ds_*-off) and for two values of the source-to-drain bias current (*I_ds_* = 0.1 and 1 µA/µm); (**b**) variation of the gate-to-source and gate-to-drain capacitance vs. *V_ds_* for three different values of the gate bias −0.3, −0.6, and −1 V).

When a transistor operates as a plasma wave detector of THz radiation in the photovoltaic mode, no DC electric current flows at its terminals; instead, a DC voltage difference, whose magnitude is direct function of the incoming optical power, sets up between the source and the drain contacts. As demonstrated by Dyakonov and Shur [13,14,15], the principle of operation of the plasma wave detection is as follows: when the device is excited by an external electromagnetic radiation, the induced AC electric fields in the channel’s device are converted into a measurable DC signal via a nonlinear conversion mechanism. Figure 6a gives the room temperature photovoltaic response of the *L_g_* = 250 nm strained-Si MODFET obtained in TCAD simulations for a frequency of 300 GHz in the photovoltaic mode (blue square symbols in Figure 6a). The photovoltaic response exhibits a maximum when the gate electrode is voltage biased at a value close to the threshold voltage of the transistor [46]. As the gate bias voltage is increased from very negative voltage values till a voltage close to the threshold one, *V_th_*, the concentration of the electron channel increases exponentially and, accordingly, the photoresponse of the transistor also rises, because the two-dimensional plasma in the channel is strengthened. For a few tens of mV of the gate overdrive, the photoresponse decays, as the carrier mobility is strongly lowered by the intense electric fields in the FET channel and electron-electron scattering in the plasma. A similar behaviour, with identical physical origin, was also observed in the roll off of the efficiency of the transconductance for positive values of the gate overdrive (Figure 5). 

**Figure 7:**
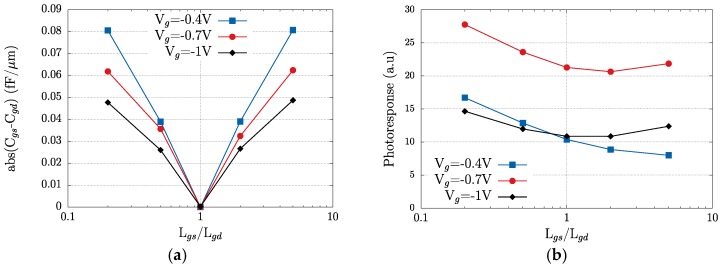
(**a**) Variation of the absolute difference of gate-to-source and gate-to-drain capacitances of the transistor as a function of the asymmetry factor *L_gs_/L_gd_*. *L_gs_*/*L_gd_* = 1 means that the gate is symmetrically disposed between source and drain contacts and *L_gs_* = *L_gd_*; (**b**) photoresponse as a function of the asymmetry factor. Simulations were performed for three gate biases (−0.4, −0.7 and −1 V).

Besides the photovoltaic mode, in Figure 6a we also show the transistor photoresponse obtained in TCAD simulations when a current bias *I_ds_* is imposed. The main effect of the use of a current bias is that THz detection is enhanced, as the photoresponse significantly grows when the bias current is increased. Since a growing DC drain current will also involve an increasing presence of electrons in the channel, this bias current will feed the plasma gas with a population of electrons supplementary to the one already supported by the gate bias voltage. Additionally, the bias current introduces further asymmetry in the channel, as the two main device capacitances split-off when the drain-to-source voltage is increased (see Figure 6b) [47,48]. The importance of introducing an asymmetry in the channel structure to enhance detection of terahertz radiation has been demonstrated [48]. The channel asymmetry can be geometrically imposed if the gate electrode is not centred between source and gate contacts, as shown in Figure 1. Figure 7a shows that modifying the position of the gate contact from a symmetric position between drain and source to a position closer to the source or the drain contacts (*L_gs_*/*L_gd_* ≠ 1) increases the difference between gate-to-source and gate-to-drain capacitances. As shown in Figure 6b, this capacitance difference will lead to an enhancement of the channel asymmetry that will help to build the photoresponse. Figure 7b gives the variation of the photoresponse with respect to the asymmetry factor (*L_gs_*/*L_gd_*) for three values of gate-to-source bias, as in Figure 7a. From Figure 7 it follows that any asymmetry enhancing the difference between gate-to-source and gate-to-drain capacitances will translate into an enhancement of the photoresponse as the gate is progressively placed closer to the source. A similar, but weaker, effect is found as the gate position approaches the drain; this increase is counterbalanced as the gate voltage is increased (blue squares in Figure 7b), as the high values of the electric field in the channel portion close to the drain contact will degrade the electron transport. 

**Figure 8:**
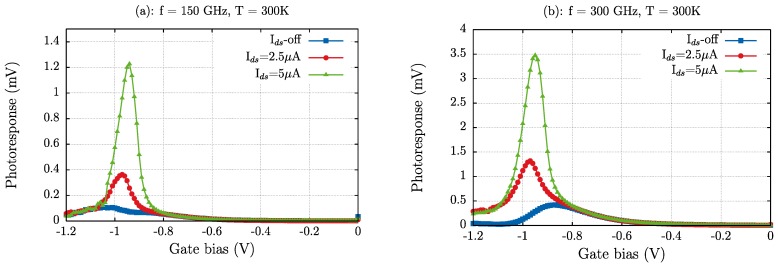
(**a**) Experimental THz photoresponse of the strained-Si MODFET in the photovoltaic mode (*I_ds_*-off) and for two values of the bias source-to-drain current (*I_ds_* = 2.5 μA, *I_ds_* = 5 μA) under an excitation of 150 GHz; (**b**) same as (**a**) under an excitation of 300 GHz.

The measured photoresponse, *ΔU*, under excitation at 150 and 300 GHz versus the gate voltage is shown in Figure 8. By comparing Figure 6a and Figure 8 it is be noticed that both measurements and simulation results exhibit the same dependence with respect to the gate bias voltage in the photovoltaic mode and, also, when a bias source-drain current is applied to the transistor. Therefore, the measured photoresponse of the strained-Si MODFET must be mainly attributed to the channel carrier action as simulations are able to essentially reproduce the experimental photoresponse both in the photovoltaic mode and when a current bias is forced into the channel. 

In the photovoltaic mode both experimental and simulation results give a photoresponse of the transistor that exhibits a maximum for a gate voltage *V_gs_ ≈ V_th_*. The behaviour of the detector response in the photovoltaic mode (a single-maximum of the intensity obtained with a gate bias voltage close to the threshold voltage of the transistor) has been reported earlier and explained as non-resonant (broadband) detection due to a low-quality factor (*Q = ωτ* < 1, *ω* being angular frequency and *τ* the electron momentum relaxation time [20], which is heavily dependent on the magnitude of the electric field and the electron concentration in the channel) associated with a low value of the electron mobility in the device’s channel. The electron channel mobility in the devices under test, while considerably higher than in conventional Si-MOSFETs (≈200 cm^2^/(V·s)), led to values of the quality factor lower than unity (assuming an average carrier mobility in the channel of about ≈1300 cm^2^/(V·s) the value of *Q* is estimated to be close to 0.13 at 150 GHz and to 0.27 at 300 GHz). Therefore, as the resonance condition is not fulfilled, the transistors exhibited a non-resonant THz detection.

After Figure 8, when a drain-to-source bias current *I_ds_* is imposed, the photoresponse grows non-linearly with *I_ds_* (an increase of *I_ds_* by a factor two leads to an increase of the maximum of the photoresponse by a factor close to three for 150 and 300 GHz). This is in agreement with the detection mechanism (plasma waves in the MODFET channel) that is modified by the forced injection of electrons in the channel by *I_ds_*, but also by the reinforcement of the channel asymmetry that non-null *I_ds_* imposes, as above discussed.

Additionally, in Figure 8 it is observed that the obtained photoresponse is more intense under excitation at 300 GHz than at 150 GHz; this must be partly attributed to the fact that the solid-state source output power at 300 GHz is twice the one at 150 GHz, and to the different role of the bonding wires and the metallic pads at 150 and at 300 GHz. Sakowicz et al. [49] have shown that for frequencies lower than 100 GHz, electromagnetic radiation is coupled to the transistor mainly by bonding wires, whereas at higher frequencies (>100 GHz) the metallization of the contact pads plays the role of efficient antennas. In fact, as it will be shown below, the transistor responsivity is similar at both frequencies, pointing to a better coupling of the incoming THz radiation to the electron channel at 150 GHz than at 300 GHz due to the wires action.

**Figure 9:**
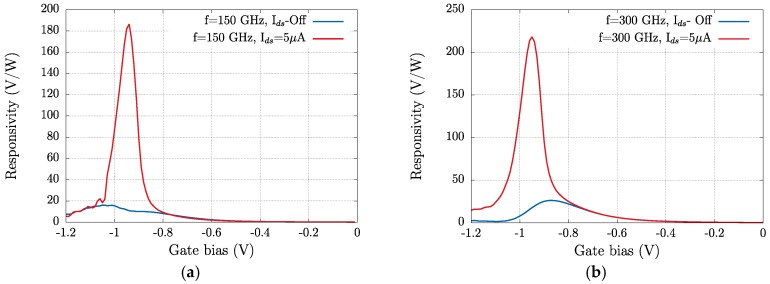
(**a**) Responsivity of the strained-Si MODFET from measurements under an excitation of 150 GHz in the photovoltaic mode (*I_ds_* off) and for a bias source-to-drain current *I_ds_* = 5 µA; (**b**) same as (**a**) under an excitation of 300 GHz.

**Figure 10:**
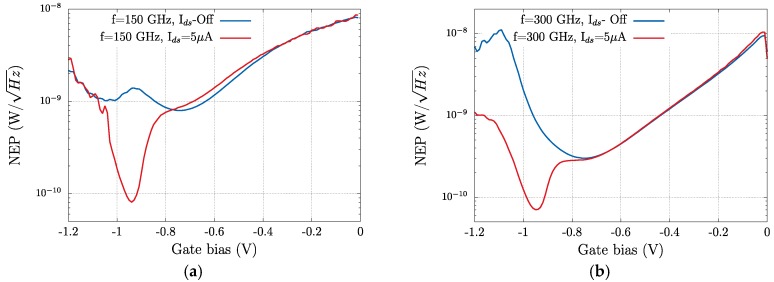
(**a**) NEP of the strained-Si MODFET from measurements under an excitation of 150 GHz in the photovoltaic mode (*I_ds_* off) and for a bias source-to-drain current *I_ds_* = 5 μA; (**b**) same as (**a**) under an excitation of 300 GHz.

Responsivity and Noise Equivalent Power (NEP) are the two key parameters—figures of merit—that determine the performance of THz detectors. The NEP is given by *N_th_*/*R_V_*, in which *N_th_* is the thermal noise of the transistor in V/Hz^0.5^ and *R_V_* is the responsivity in V/W. Since detection is studied at zero drain bias, the thermal noise *N_th_* = (4*kTR_ds_*)^0.5^ is the only relevant source of noise of the transistor. Here, *R_ds_* is the drain-to-source resistance that can be extracted from the transfer characteristics measured at low drain bias (20 mV) corresponding to the linear regime (Figure 4). Figure 9 and Figure 10 present, respectively, the dependence of *R_v_* and NEP on the gate bias voltage.

The responsivity is calculated according to the expression:

(6) $$R_{V}=\frac{{\Delta \mathrm{US}}_{t}}{P_{t}S_{a}}\frac{\pi }{\sqrt{2}},$$

in which Δ*U* is the measured photoresponse, *S_t_* is the radiation beam spot area, *S_a_* the active area of the transistor, and *P_t_* the total incident power at the HEMT detector position. The radiation beam power at the detector position was measured using a calibrated pyroelectric detector; the *P_t_* values were *P_t_* = 0.5 mW at 150 GHz and *P_t_* = 1 mW at 300 GHz. The spot area is given by *πr^2^*, in which *r* is the radius of the beam spot (≈1.5 mm at 300 GHz and 3.3 mm at 150 GHz). The area of the transistor, including the contact pads, is lower than 0.05 mm^2^ (Figure 1), which is much smaller than the diffraction limit area *S_λ_ = λ^2^/4*. An accurate determination of the active area of the detector is difficult, as also the bonding wires and/or the contacts pads may partially play an antenna role; therefore, although the choice will lead to an underestimation of the transistor responsivity, it is advisable to select *S_λ_* as the active area. Accordingly, to calculate *Rv* in Equation (6), *Sa* was replaced by *S_λ_*. The factor π/√2 originates from the Fourier transform of the square wave modulated THz signal detected as rms value with a lock-in.

The responsivity of the transistor is greatly enhanced by the imposed drain-to-source bias current at both frequencies. As above stated, the noticeable difference found in the magnitude of the photoresponse at 150 and 300 GHz (Figure 8) is very modest in terms of the responsivity of the device. Table 1 summarizes the extreme values of the two figures of merit studied; the maximum value of *R_V_* (*R_v_*_,*max*_) is enhanced by near one order of magnitude when a 5 µA drain-to-source bias current is present. Additionally, the minimum value of the NEP at both frequencies is significantly lowered when the drain-to-source bias current is imposed. As it is presented in Figure 9, the NEP reduction induced by the bias current takes place, essentially, when the device operates close to or in the subthreshold region. This is consistent with the extra electron population in the channel supported by the bias current that reduces *R_ds_* (channel resistance) and the minimum value of NEP (NEP_min_). The intensity of the applied current is kept low to avoid noise increase in the measurements.

**Figure 11:**
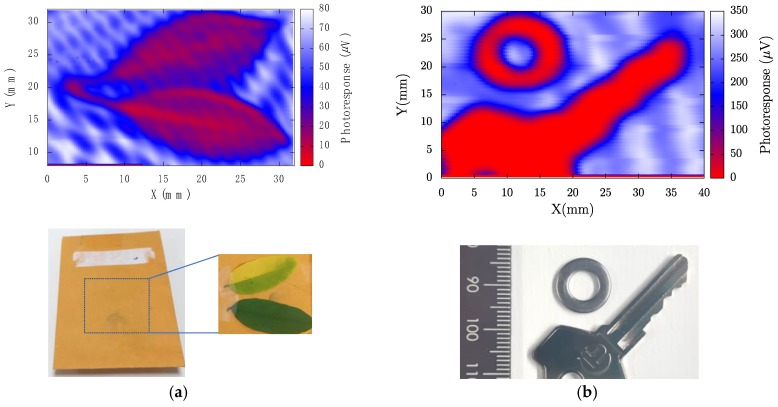
(**a**) 300 GHz image of two leaves inside an envelope and, below, photograph of the envelope showing the position of the leaves; (**b**) 300 GHz image of two metallic objects inside an envelope and, below, photograph of the objects.

To test the ability of the strained-Si MODFETs as detectors in THz imaging, a single transistor was used as the sensor in the terahertz imaging system of Figure 3. More information about the terahertz imaging system setup can be found in [31]. The radiation passes through the hidden object, and the intensity is measured by the strained-Si MODFET that is biased around the threshold voltage to obtain a maximum intensity of the signal. Figure 11 shows the terahertz images of organic and metallic objects obtained under illumination of THz radiation at 300 GHz along with their images in the visible spectrum. The clear terahertz images obtained from hidden objects confirm the suitability of strained-Si MODFETs as detectors to build high-quality THz images. 

## 4. Conclusions

Plasma waves in gated 2-D systems can be used to efficiently detect THz electromagnetic radiation. Solid-state plasma wave-based sensors can be used in THz imaging systems. An experimental study of the sub-THz response of II-gate strained-Si Schottky-gated MODFETs (Modulation-doped Field-Effect Transistor) was presented. As the photoresponse of any plasma wave FET detector depends on many parameters (the whole device topology, the material system and the excitation frequency) to understand its THz response, a two-dimensional hydrodynamic-model was used. 

The response of the strained-Si MODFET has been characterized at two frequencies: 150 and 300 GHz: The DC drain-to-source voltage transducing the THz radiation (photovoltaic mode) of 250-nm gate length transistors exhibited a non-resonant response that agrees with theoretical models and TCAD simulations. When imposing a weak source-to-drain current of 5 μA, we found an increase of the photoresponse that is translated into an enhancement of the responsivity by one order of magnitude as compared to the photovoltaic mode, while the NEP is contained to/improved by a factor lower than three. Strained-Si MODFETs demonstrated an excellent performance as detectors in THz imaging.

## Figures and Tables

**Table 1:** Figures of merit of the strained MODFETs as THz detectors.

Frequency (GHZ)	*I_ds_* (μA)	*R_V,max_* (V/W)	NEP_min_ (pW/√Hz)
150	0	16	320
150	5	186	81
300	0	26	300
300	5	218	70
